# Fenofibrate Therapy in Carnitine Palmitoyl Transferase Type 2 Deficiency

**DOI:** 10.1155/2012/163173

**Published:** 2012-12-03

**Authors:** I. Hamilton-Craig, M. Yudi, L. Johnson, R. Jayasinghe

**Affiliations:** ^1^Griffith University School of Medicine and Gold Coast University Hospital, Southport, Gold Coast, QLD 4215, Australia; ^2^Royal Australasian College of Physicians and Royal Melbourne Hospital, Parkville, Melbourne, VIC 3050, Australia; ^3^Pathology Queensland, Royal Brisbane and Women's Hospital, Brisbane, QLD 4029, Australia; ^4^Department of Cardiology, Gold Coast University Hospital, Griffith University, Southport, Gold Coast, QLD 4215, Australia

## Abstract

Bezafibrate therapy has been shown to improve beta-oxidation of fatty acids and to reduce episodes of rhabdomyolysis in patients with carnitine palmitoyltransferase type-2 (CPT2) deficiency. We report the efficacy of fenofibrate in a patient with CPT2 deficiency, in whom beta-oxidation was improved but an episode of rhabdomyolysis nevertheless occurred. This suggests additional methods to avoid rhabdomyolysis in patients with CPT2 deficiency should accompany fibrate therapy, including avoidance of muscular overexertion, dehydration, and heat exposure.

## 1. Introduction

Carnitine palmitoyl transferase type 2 (CPT2) deficiency is a rare autosomal recessive disorder of mitochondrial fatty acid oxidation [1]. It manifests clinically by muscle stiffness, myalgia, exercise intolerance, and episodes of rhabdomyolysis especially after overexertion, heat exposure, viral infection or other intercurrent illness [1]. 

In 2009, Bonnefont and colleagues found that bezafibrate therapy restored the capacity for normal fatty acid oxidation in muscle cells from patients with CPT2 deficiency by stimulating the expression of the mutated gene [2]. They described a series of six patients with CPT2 deficiency treated with bezafibrate 200 mg three times daily for a period of 6 months. Bezafibrate therapy resulted in 60–284% improvement in skeletal muscle palmitoyl L-carnitine oxidation levels, 20–93% increase in skeletal muscle CPT2 mRNA, and full correction of the initial defective fatty acid oxidation in myoblasts *in vitro*. The number of episodes of rhabdomyolysis was reduced from 3–24 per patient over a 6-month period before treatment to 0–6 per patient over a 6-month period after treatment. Levels of creatine kinase (CK) were reduced from a mean of 10,900 IU/L before treatment to 4,700 IU/L after treatment, indicating a reduced rate of muscle damage [2].

Unlike gemfibrozil and fenofibrate, bezafibrate is not widely available in many countries, necessitating therapy with the former two agents for patients with dyslipidaemia, the common indication for fibrate use. 

In this paper we report the case of a patient with CPT2 deficiency who responded to fenofibrate therapy with regard to improvement in plasma acylcarnitine and lipid levels, as well as improvement in muscle symptoms. While on fibrate therapy, he nevertheless experienced an episode of rhabdomyolysis after heat exposure and a viral infection. As in the experience of Bonnefont et al., fibrate therapy did not completely abolish episodes of rhabdomyolysis in this patient in spite of evidence for improved CPT2 activity. Therefore, it appears that traditional means to prevent rhabdomyolysis in CPT2 deficiency such as avoidance of overexertion and dehydration continue to be required during fibrate therapy for this rare condition. 

## 2. Initial Presentation

J. W., a 59-year-old immigration officer, was referred by his general practitioner for private specialist management of dyslipidaemia. From a young age, J. W. had experienced virtually constant muscle stiffness and recurrent episodes of severe muscle aches and pains. His past medical history included hypertension treated with lisinopril 20 mg daily for 5 years. A diagnosis of CPT2 deficiency was made by histochemical analysis of a muscle biopsy at the age of 39 years. Mutation analysis is yet to be performed. His only sibling also had similar symptoms, with a presumptive diagnosis of CPT2 deficiency in the absence of a muscle biopsy, which she has refused. There was no other family history suggestive of CPT2 deficiency. On examination, the body mass index was 28.55 (height 189 cm, weight 102 kg), waist circumference 105.5 cm, blood pressure 123/78, and pulse rate 64 and regular. There was no evidence of arcus senilis, or xanthoma. He belonged to the intermediate risk category for a coronary event over the next 5 years (10–15% risk) according to current Australian cardiovascular risk calculator [3].

In view of his intermediate risk, the presence of subclinical atherosclerosis was investigated with coronary artery calcium score (CACS) and carotid duplex ultrasonography [4]. There was a calcified plaque in the distal segment of the left main coronary artery. The CACS was 90 Agatston units with volume 68 mm^3^, between the 50th and 75th percentile for asymptomatic men of the same age [5]. Subsequent stress echocardiography was normal. Duplex carotid ultrasonography revealed generalised intimal thickening without isolated carotid plaque formation. The maximum intima-media thickness was 2.0 mm in the distal walls of the midcommon carotid arteries (CCA). Flow parameters were within the normal range and the vertebral artery flow was antegrade bilaterally.

Fasting lipids were remeasured. Levels of total cholesterol (TC), low-density lipoprotein cholesterol (LDL-C), high-density lipoprotein cholesterol (HDL-C), and triglycerides (TG) were 7.4, 4.4, 1.5, and 1.5 mmol/L, respectively (Table 1). Levels of CK (70 U/L, *R* < 190), TSH (2.8 U/L, *R* 0.4–4.0), and Lp (a) (<80 mg/L, *R* < 300) were normal. Plasma homocysteine was elevated (26.8 umol/L, *R* 0–15.0). ApoA1 (2.1 g/L, *R* 1.0–2.0) and ApoB100 (1.3 g/L, *R* 0.6–1.7) were normal. The urate level was increased (0.65 mmol/L, *R* 0.15–0.50). Plasma acylcarnitine levels were measured by ultraperformance liquid chromatography electrospray ionization tandem mass spectrometry [6]. Acylcarnitine levels were typical of CPT2 deficiency [7] with grossly elevated levels of palmitoylcarnitine (1.6 umol/L, *R* < 0.65 umol/L); see Table 1. Plasma levels of total and free carnitine were within the normal range (Table 1).

In view of the finding of elevated LDL-C levels and significant subclinical atherosclerosis, J. W. was treated initially with low-dose rosuvastatin 5 mg daily in view of his underlying metabolic myopathy [8, 9]. A target LDL-C of <2.0 mmol/L was defined in view of his increased CVD risk [8]. He was advised to report aggravation of muscle symptoms and to immediately cease therapy should this occur. He was also treated with allopurinol 300 mg daily for the elevated urate level and folate 5 mg daily to reduce homocysteine levels.

Two months later his lipid profile had improved considerably to TC 4.9 mmol/L, LDL-C 2.5 mmol/L, HDL-C 1.5 mmol/L, and TG 1.1 mmol/L (Table 1), but the CK level had increased to 1400 U/L. There was no significant change in myalgia symptoms, and the patient admitted to working in the garden for 1-2 days before the blood test. Rosuvastatin was ceased and replaced with fenofibrate 145 mg daily plus ezetimibe 10 mg daily. 

Within 8 weeks, he complained of increasing myalgia and a skin rash. The CK level was 250 U/L (*R* < 190). Ezetimibe was ceased and fenofibrate continued with resolution of the rash and of symptoms to pretreatment levels. After 6 months of fenofibrate therapy, acylcarnitine levels showed evidence of improved beta-fatty acid oxidation (presumably as a result of increased CPT2 activity), as observed for bezafibrate therapy in patients with CPT2 deficiency by Bonnefont et al. (Table 2) [2, 10, 11]. The level of palmitoylcarnitine had fallen from 1.6 to 0.3 umol/L and other acylcarnitines had improved significantly. Total and free carnitine levels remained within the normal reference range (Table 2). During this period, J. W. reported improvement in general subjective musculoskeletal symptoms, with less stiffness and fewer aches and pains. The subjective average levels of these symptoms were reduced by about 50% from 4–6/10 in severity prior to fenofibrate therapy to 2-3/10 in severity after fenofibrate therapy. Objective measurement was not performed. His cardiac status remained stable with no new angina or other symptoms of concern; objective cardiac testing was not performed.

Four months later, J. W. went on holiday and spent considerable time exposed to the sun while undertaking vigorous snorkelling and swimming. The day after returning from his holiday he had an upper respiratory tract infection followed one day later by mild generalised muscle aches and cramping. Within 48 hours his urine became dark and he complained of severe generalised muscle pain and stiffness. He was admitted to the local hospital with rhabdomyolysis. Blood levels on admission were CK 34,600 (*R* < 190), aspartate transaminase (AST) 1450 (*R* < 35 U/L), and alanine transaminase (ALT) 465 (*R* < 45 U/L). 

In hospital all medications were ceased and he was rehydrated with rapid resolution of symptoms. He was discharged after 3 days, when his blood levels were CK 11,300 U/L, urea 7.7, creatinine 123., eGFR 52, AST 937, and ALT 465. Followup was arranged with the hospital's renal outpatient department. 

Twenty-three months after the initial consultation, and two months after cessation of all lipid-lowering therapy during admission for rhabdomyolysis, J. W.'s lipid and acylcarnitine profiles had returned to pretreatment levels (Table 2). His renal function remained impaired (eGFR 34 mL/min, *R* > 59; creatinine 178 umol/L, *R* 60–140). The urate level was also elevated (0.5 mmol/L, *R* 0.12–0.45). Fenofibrate, allopurinol, and lisinopril therapies were recommenced at doses of 96 mg, 300 mg, and 5 mg daily, respectively, with subsequent improvement in urate level, and lipid and acylcarnitine profiles (Tables 1 and 2). Levels of CK subsequently remained normal. No further episodes of rhabdomyolysis have occurred, and muscle symptoms have remained stable (with variable stiffness and mild aching, average severity 2–4/10). No new cardiac symptoms of concern have occurred.

## 3. Discussion

The case of J. W. raises a number of general issues with regard to the management of dyslipidaemia in patients with subclinical atherosclerosis and musculoskeletal side effects of therapy, in addition to specific issues related to CPT2 deficiency. 

The general principles for management of hyperlipidaemia were adhered to, namely, repeating the lipid profile if abnormal, excluding secondary causes of hyperlipidaemia, and maintaining a lipid-lowering diet as the foundation of therapy. However, drug therapy was not commenced at a dose likely to achieve target LDL-C levels (<2.0 mmol/L). Instead, a lower dose (5 mg rosuvastatin) was used in view of the known history of CPT2 deficiency and the propensity of any underlying myopathy to be aggravated by statin therapy [8, 9]. This dose of rosuvastatin was able to achieve a near-target LDL-C of 2.5 mmol/L (42% reduction from baseline). It was decided to withdraw rosuvastatin because of statin-associated myalgia with an elevated CK level >3 × ULN [8]. 

Aggravating and precipitating factors for myalgia should then be sought, including overexertion, hypothyroidism, low vitamin D levels, and drugs that can interfere with statin catabolism such as cyclosporine, macrolide antibiotics, and antifungals [8, 9]. Once the CK has returned to normal, and symptoms have resolved, it may be possible to reintroduce statin either in a lower dose, or with an alternative drug of the class. If statin is not tolerated, or if the tolerated dose is insufficient to achieve target LDL-C levels, ezetimibe is the next-line drug of choice for use (extended-release niacin would be an alternative but is not available in Australia). Bile acid sequestrants are not commonly used because of poor compliance. Coenzyme Q10 supplements were not used as supporting evidence is insufficient for this therapy to be included in guidelines [9].

It was decided to treat J. W. with fenofibrate therapy in view of the data of Bonnefont et al., showing subjective and objective improvement in lipid and CPT2 parameters with bezafibrate therapy [2, 11]. In order to achieve further LDL-C reduction and achieve target levels, ezetimibe was initially coadministered with fenofibrate. This achieved an LDL-C level of 2.4 mmol/L, similar to that achieved with rosuvastatin 5 mg daily (Table 1). Ezetimibe was subsequently withdrawn because of aggravation of myalgia and skin rash–both uncommon but documented adverse effects of ezetimibe. Fenofibrate monotherapy then achieved variable LDL-C levels between 2.35 and 3.4 mmol/L, compared with untreated levels between 4.3 and 5.2 mmol/L (Table 1).

With regard to acylcarnitine levels, therapy with fenofibrate resulted in significant improvement, particularly for oleoylcarnitine (95% reduction) and palmitoylcarnitine (80% reduction). This suggests improved mitochondrial beta-oxidation of fatty acids through increased CPT2 activity, consistent with previous results of Bonnefont et al. with bezafibrate [2, 11]. In spite of this improvement, however, J. W. experienced an episode of rhabdomyolysis whilst on fenofibrate therapy, suggesting insufficient fatty acid oxidation in the presence of mitochondrial stress arising from heat exposure, dehydration, or intercurrent illness such as a viral infection. 

The acylcarnitine results (Table 2) were obtained in the fasting state, with no previous undue exertion or evident infection, and the patient was on a stable cholesterol-lowering diet with restricted intake of saturated fats and cholesterol. Nevertheless, some variation in acylcarnitine levels could have been caused by variation in environmental factors (including dietary changes, fasting versus postabsorptive state, physical exertion, and infection) in addition to lipid-lowering therapy.

Our data when considered with those of Bonnefont et al. suggest a class effect for fibrates in improving CTP2 activity, and a potential use for fibrates in patients with CPT2 deficiency [2, 10, 11]. There are some differences in structure, pharmacodynamics, and PPAR-alpha agonist activity between bezafibrate and fenofibrate (12). Whether one fibrate is preferable to another in patients with CPT2 deficiency requires further investigation.

## 4. Summary and Conclusions

It is important to achieve optimal LDL-C reduction in asymptomatic patients with significant asymptomatic atherosclerosis [8]. A target LDL-C <2.0 mmol/L is generally accepted, with no lower limit so long as adverse effects do not occur. Patients intolerant of statins represent an important group because they are not uncommon in clinical practice and often fail to achieve satisfactory LDL-C reduction and consequently remain at increased CVD risk. 

Muscle adverse effects are the most frequently encountered adverse effect of statin therapy, and the most common cause for statin intolerance. Patients with underlying metabolic myopathies are particularly prone to statin muscle adverse effects for reasons that remain unclear, as the mechanism(s) of statin myopathy are currently poorly defined and are probably multifactorial. Other patients prone to statin myopathies are those with hypothyroidism and vitamin D deficiency, in whom replacement of thyroxine or vitamin D usually ameliorate the adverse effects of statins. 

There have been few reports of statin adverse effects in patients with CPT2 deficiency, but J. W.'s experience with increased CK levels and muscle symptoms was not unexpected and necessitated withdrawal of statin therapy. Whether lower statin doses in patients with statin myopathy (e.g., rosuvastatin 2.5 mg three times weekly, as used currently by many investigators with moderate success) would have been successful in J. W. remains problematic. The use of additional agents such as bile acid sequestrants and/or niacin could be considered in future, but these agents are generally poorly tolerated. 

Another important aspect of J. W.'s management is monitoring of subclinical atherosclerosis, which is likely to progress in the presence of an elevated LDL-C and renal impairment. Annual carotid ultrasonography and stress echocardiography is planned, with repeat calcium scoring after 2-3 years to assess the rate of progression of subclinical coronary atherosclerosis. Hopefully means to lower LDL-C currently in development will be available in Australia in the near future. These include mipomersen (apoB100 silencing RNA therapy, currently undergoing phase III trials for patients with familial hypercholesterolaemia) and triple therapy with niacin-laropiprant-simvastatin (as available in Europe).

## Figures and Tables

**Table 1:** Change in lipid levels with therapy.

Months	Lipid therapy (mg/d)	TC*	TG*	HDL-C*	LDL-C*
0	Nil	7.4	1.5	1.5	4.4
1	Nil	7.1	1.4	1.6	4.3
4	Rosuvastatin 5 mg	4.9	1.1	1.5	2.5
7	Fenofibrate 145 mg + ezetimibe 10 mg	5.0	0.8	1.6	2.35
11	Fenofibrate 145 mg	5.9	1.0	1.7	3.1
17	Fenofibrate 145 mg	5.4	1.5	1.3	3.4
19	Fenofibrate 145 mg	5.0	0.8	1.6	2.35
22	Fenofibrate 145 mg	5.9	1.0	1.7	3.1
28	Nil	7.6	3.7	1.3	4.8
30	Nil	7.4	1.7	1.4	4.8
31	Nil	7.9	1.8	1.2	5.2
33	Fenofibrate 96 mg	6.1	1.5	1.6	3.4

*TC: total cholesterol, TG: triglycerides, HDL-C: high density lipoprotein cholesterol, LDL-C: low density lipoprotein cholesterol (mmol/L).

**Table 2:** Changes in acylcarnitine levels (umol/L) with therapy.

Months	Lipid therapy	C16:0 (*R* ^#^ < 0.65)	C18:0 (*R* 0.02–0.10)	C18:1 (*R* < 0.02)	C18:1-OH (*R* ^#^ n/a)	C2:0 (*R* 3–23)	C3:0 (*R* < 0.66)	C total (*R* 21–70)	C free (*R* 13–56)
1	Nil	1.6	0.84	2.64		21	n/a	95	66
7	Fenofibrate 145 mg + Ezetimibe 10 mg	0.3	0.46	0.14		n/a	0.71	105	91
17	Fenofibrate 145 mg	1.3	1.34	0.72	0.02	13	0.71	86	68
30	Nil	1.8	2.6	0.8	0.80	20	0.80	104	76
33	Fenofibrate 96 mg	1.2	0.44	1.46	n/a	20	0.79	145	119

^#^ *R*: provisional reference interval (umol/L).

C16:0: palmitoylcarnitine; C18:0: stearoylcarnitine; C18:1: oleoylcarnitine; C2:0: acetylcarnitine; C3:0: propionylcarnitine; C18:1-OH: hydroxyoleylcarnitine; C total: total carnitine; C free: free carnitine.
